# HIV-associated neurocognitive disorders

**DOI:** 10.1186/2049-9256-2-2

**Published:** 2014-03-04

**Authors:** Montserrat Sanmarti, Laura Ibáñez, Sonia Huertas, Dolors Badenes, David Dalmau, Mark Slevin, Jerzy Krupinski, Aurel Popa-Wagner, Angeles Jaen

**Affiliations:** Servei de Medicina Interna, Unitat VIH/Sida, Hospital Universitari MútuaTerrassa, Pl.Dr.Robert, 5, 088221 Terrassa, Barcelona, Spain; Fundació Docència i Recerca MútuaTerrassa, Terrassa, Barcelona, Spain; Servei de Neurologia, Hospital Universitari MútuaTerrassa, Barcelona, Spain; Department of Healthcare Science, Manchester Metropolitan University, Manchester, UK; Clinic for Psychiatry and Psychotherapy, University of Medicine Rostock, Rostock, Germany

**Keywords:** HIV, Neurocognitive disorders, HAND

## Abstract

Currently, neuropsychological impairment among HIV+ patients on antiretroviral therapy leads to a reduction in the quality of life and it is an important challenge due to the high prevalence of HIV-associated neurocognitive disorders and its concomitant consequences in relation to morbidity and mortality- including those HIV+ patients with adequate immunological and virological status. The fact that the virus is established in CNS in the early stages and its persistence within the CNS can help us to understand HIV-related brain injury even when highly active antiretroviral therapy is effective. The rising interest in HIV associated neurocognitive disorders has let to development new diagnostic tools, improvement of the neuropsychological tests, and the use of new biomarkers and new neuroimaging techniques that can help the diagnosis. Standardization and homogenization of neurocognitive tests as well as normalizing and simplification of easily accessible tools that can identify patients with increased risk of cognitive impairment represent an urgent requirement. Future efforts should also focus on diagnostic keys and searching for useful strategies in order to decrease HIV neurotoxicity within the CNS.

## Review

Disruption of neurocognitive functioning is one of the most frequent complications in patients infected with HIV nowadays. This is a common reason for consultation of HIV patients and it negatively affects their quality of life, treatment adherence and life span. Since the introduction of Highly Active Antiretroviral Therapy (HAART) the spectrum of HIV-associated neurocognitive disorders (HAND) has been radically changed with a significant reduction in dementia but with a high prevalence of asymptomatic and mild neurocognitive impairments. Currently, in clinical practice, patients with HIV are still admitted to the clinic for memory, concentration or planification problems even when virology is under control. These usually begin with subtle changes but it can lead to more severe forms of neurocognitive impairment. The aim of this review is to describe the different types of neurocognitive disorders, possible mechanisms of development, epidemiology and risk factors in HIV patients, as well as the clinical approach and current treatment of HAND.

### Classification

Before 1991, there was only one kind of neurocognitive disorder defined, the HIV-associated dementia (HAD), which was known as the complex AIDS-Dementia. It affected patients with severe immune-depression causing severe impairment of cognition, frequently accompanied by motor and behavioural alterations. More recently, the American Academy of Neurology proposed a new classification by defining two levels of neurological impairment in patients with HIV: the classical HAD and the minor cognitive motor disorder (MCMD) representing patients that did not meet dementia criteria but complained of slight impairments that interfered with their daily life [1].

In 2007, a further revised classification system of HAND was introduced which is thought to be more precise and sensitive (Frascati Criteria). It describes, beside HAD, other two neurocognitive disorders: Mild Neurocognitive disorder (MND) and Asymptomatic Neurocognitive impairment (ANI). MND is defined as mild to moderate impairment within at least two cognitive areas with at least mild impairment of daily function. ANI is defined as any degree of neuropsychological testing impairment in at least two cognitive domains but without causing an observable functional impairment [2] (See the “Classification of HAND” Section). Finally, cognitive neuropsychology aims to elucidate the component processes of HAND across the domains of executive functions, motor skills, speeded information processing, episodic memory, attention/working memory, language, and visuoperception [3].

### Classification of HIV-associated neurocognitive disorders

Asymptomatic neurocognitive impairment (ANI)

1. No evidence of preexisting cause. Cognitive impairment must be attributable to HIV and no other etiology (e.g. dementia, delirium).
2. The cognitive impairment does not interfere with activities of daily living.
3. Involves at least two cognitive areas (memory, attention, language, processing speed, sensory- perceptual, motor skills) documented by performance of > 1 standard deviation below the mean of standardized neuropsychological testing.

Mild neurocognitive disorder (MND)

1. No evidence of preexisting cause. Cognitive impairment must be attributable to HIV and no other etiology (e.g. dementia, delirium).
2. At least mild interference in > 1 activities of daily living including mental acuity, inefficiency at work, homemaking or social functioning.

HIV-Associated dementia (HAD)

1. No evidence of another preexisting cause for dementia (i.e. CNS infections, CNS neoplasm, cerebrovascular disease).
2. Marked interference in activities of daily living.
3. Marked cognitive impairment involving at least two cognitive domains by performance of > two standard deviation below the mean of standarized neuropsychological tests, especially in learning of new information, slowed information processing and defective attention or concentration.

### Neuropathogenesis

The reason for neurocognitive disorders in HIV-patients is still unclear. It is well known that the central nervous system (CNS) is one of the target organs where HIV can be detected soon after primary infection. Early neuro-invasion is characterized by measurable markers of CSF inflammation (eg, neopterin level) and by brain parenchymal inflammation detected by magnetic resonance spectroscopy (MRS), although changes in neurocognitive functioning are seen more clearly in advanced stages [2, 4]. HIV enters the brain carried within migrating monocytes and lymphocytes that cross the blood brain barrier (BBB) and possibly CD4+ T lymphocytes that use the brain as a reservoir for viral replication. After crossing the BBB, HIV infected monocytes become active perivascular macrophages being able to produce HIV within the CNS, release free virions and facilitate infection of microglial cells [5]. Both cells types allow HIV replication concomitant with expression of neurotoxic molecules- thought to be the ones involved in the pathogenesis of HAND [6] (eg. soluble immune mediators). Astrocytes may also harbour HIV and contribute to HIV-related brain disease through mechanisms of astrogliosis induced by local chemokines and cytokines leading to increased BBB permeability and consequently, monocyte and lymphocyte migration. Neurons are not productively infected.

Autopsy studies of AIDS patients with HAD shows characteristic white matter changes and demyelination, microglial nodules, multinucleated giant cells and perivascular infiltrates [7]. A variety of proteins encoded by the viral genome were identified as neurotoxic agents, including gp120, the virus’s envelope protein, and transactivator of transcription (Tat). Gp120 is necessary for infectivity, but also interacts with host cellular receptors to alter glutamate pathway signaling and induce cytokine production that can injure neurons and affect the activation state of microglia and astrocytes.

Tat protein which is produced by infected astrocytes [8] has both neurotoxic and proinflammatory features. In several studies where Tat-expressing astrocytes were injected into the rat dentate gyrus it caused dendritic loss and neuronal cell death [9]. Furthermore, it is known that the virus is not evenly distributed in the CNS. Different areas of the brain are more susceptible to HIV due to the presence of individual viral genetic conditions and characteristics of the host [10]. The regions in which HIV is most prevalent are basal ganglia and the hippocampus, and to a lesser extent in the cortex and cerebellar grey matter of the mid-frontal cortex. These findings correspond with neuropsychological impairment of the fronto-subcortical-region and dysfunction, corresponding to frontal and subcortical areas [11]. Patients infected with HIV frequently have co-morbid conditions that may contribute to or amplify the pathogenicity of HIV, thus having an important role in the development of neurocognitive impairment (e.g. drug abuse and alcohol or infection with viral co-pathogens such as hepatitis C virus) [12].

Other factors that may contribute to HAND are the prolonged survival of HIV-infected patients, thereby extending the brain’s exposure to HIV virions and proteins, the use of increasingly toxic combinations of poorly penetrating drugs in highly antiretroviral-experienced AIDS patients, and selection of more virulent HIV strains with higher replication rates and greater virulence in neural tissues. Eden and colleagues found that a substantial number of patients still show signs of macrophage/microglia activation even after 4 years of viral suppression with HAART [13]. All these factors converge in producing damage to dendrites and synapses leading to the disruption of the highly integrated functioning of neural systems that is required to process information, leading to HIV-associated neurocognitive disorders.

### Epidemiology

Several studies demonstrated that neurocognitive deficits are more common in HIV+ patients than the HIV- population, and these deficits were independent of either ART, or disease state [14, 15]. However, one recent study demonstrated similar rates of cognitive impairment between a subgroup of HIV+ patients, with a high CD4 level and HIV viral suppression as compared with HIV- controls [16]. A high prevalence of HIV-associated neurocognitive disorders (HAND) is associated with a change in the pattern of HAND and with an increased prevalence of mild disease vs dementia [17, 18]. Before the introduction of HAART, the HIV-associated neurocognitive impairment was categorized as AIDS-dementia complex, HIV encephalitis or encephalopathy, with an estimated prevalence of 16% in patients with a diagnosis of AIDS [19]. However, in the post-HAART era, the prevalence of dementia is estimated to be less than 5% [17, 20]. In contrast, it appears that mild cognitive disorders are very common despite treatment [17].

The prevalence of HAND is estimated in approximately 40-50% of all cases. Mild neurocognitive disorder (MND) and Asymptomatic Neurocognitive Impairment (ANI) are now more common than HAD. In a cross-sectional study conducted in Switzerland, there was a prevalence of HAND in 85% of patients with evidence of cognitive decline, and 64% among those who did not demonstrate any deterioration. In the first group, 24% had ANI, 52% MND and 8% HAD, while in the second group 60% had ANI, 4% MND and 0% HAD. Subclinical alterations that do not affect the functionality of the patients or alter their daily lives are the most frequent in both groups [17]. In a cross-sectional study conducted on 1555 HIV infected patients the CNS HIV Anti-Retroviral Therapy Effects Research (CHARTER) found that 52% had HAND, of which 33% had ANI, 12% MND and 2% HAD. The prevalence of impairments correlated with the number of patient comorbidities [20]. Another study showed a 48% rate of occurrence of cognitive decline in HIV-infected patients [21], whilst the Aquitaine cohort found a prevalence of 58.5% TNAVs, 20.8% ANI, 31% MND, and 6.7% HAD [18]. In Spain, a very recent multicenter study conducted by questionnaire, found that 50% of the HIV+ population complained of cognitive impairments. A high proportion of them (72.1%) believed that their cognitive impairments interfered with daily life activity [22].

#### Progression and implication

The real clinical relevance of ANI is not clear. It does not interfere with daily life and in addition, it is not proven that ANI patients are at increased risk of progression to more advanced stages of deterioration. Moreover, there are no satisfactory biomarkers or clinical prognostic/diagnostic indicators associated with this subgroup of patients that can determine evolution and end-point. The CHARTER study reported preliminary results of monitoring at 18 and 42 months. The proportion of patients with worsening neurocognitive function according to their basal HAND classification was different in each group (ANI-23%, MND-30%, not TNAV-13%), however there was no statistically significant difference in mean decline in neurocognitive function between ANI and MND groups [23]. From a prospective cohort, ACTG Longitudinal Linked Randomized Trials (ALLRT) of 1160 patients from clinical trials, 921 were examined using neuropsychological tests, and were subsequently followed for 48 weeks. The prevalence of HAND was 39% at inclusion, of them 44% improved after one year. In contrast, of those patiens without HAND, 21% developed worse cognitive function [24].

### Risk factors

The risk factors associated with neurocognitive disorders are not well established in the post HAART era. They may include host factors (e.g. genetic predisposition, metabolic disorders, cardiovascular risk factor or aging), HIV-related factors (AIDS, immune activation, drug resistance) and comorbidities such as hepatitis C virus co-infection or depression. Poor immunological status, reflected by lower nadir CD4 cell count, has been associated with neurocognitive impairment before and after HAART [20, 24, 25]. However, other studies did not find this association with the CD4 levels [17, 18, 26]. The correlation with virological status is even less clear in the HAART era [17, 18, 26]. Older age was associated with neurocognitive impairment [18, 21, 25] and HAD [27]. Valcour and collaborators [27] found about 3-fold increased risk of HAD in patient >50 years compared with those between 20 to 39 years old, independently of other factors. Harezlak and co-workers found an association between age and markers of brain injury in HIV infected patients as compared with those being HIV negative [21]. However, in other studies there was not a higher age-related impairment of neuropsychological function between HIV+ and HIV- populations [28]. Comorbidities, such as coinfection with hepatitis C virus (HCV), were found to be a risk factor for the presence of HAND [25, 29] that could be explained by an independent neurotoxic effect of HCV. Other comorbidities as obesity and diabetes were associated with neurocognitive impairment, especially in older patients [30].

Data on neuropsychiatric symptoms in coinfected persons are inconclusive at this time. With regard of depression was the most common disorder by far, reaching rates of 71% and 62% for past depression in coinfected and HIV-infected persons, respectively, and 42% for current rates in both groups. Past dysthymia, past posttraumatic stress disorder, and childhood conduct disorder were the next most prevalent, with rates for each ranging between 16% and 19% in both groups [31]. Other cardiovascular risk factors and diseases were associated with lower cognitive performance [18, 32]. Host genetic factors such as apolipoprotein E4 have been suggested as possible risk factors for neurocognitive impairment [33]. Whilst in the general population, sub-groups with the ApoE4 allele have a worse prognosis for neurodegeneration [34], this risk is less clear in the HIV+ population [35, 36].

### Clinical features

The landmark of neuropsychological impairment in patients infected with HIV has changed in recent years particularly after the introduction of HAART. Currently, most patients with alterations in neurocognitive tests have no evident symptoms or dysfunction in their daily lives (ANI). It has been demonstrated that the neurocognitive impairment in early stages of infection shows a subcortical pattern and in more advanced stages implicates more cortical areas. The main cognitive complaints of the patients with mild cognitive dysfunction involve subtle changes in working memory and attention, speed of informational processing, executive functioning (including organizing, planning and problem-solving) or difficulties in verbal fluency. Motor symptoms are less common but can include leg weakness, tremor or unsteady gait. HAD, however, is often characterized by a progressing subcortical dementia with classically severe memory loss and altered executive function. More advanced stages also show aphasia, agnosia and apraxia, that are more typical of cortical dementias such as Alzheimer’s disease. Other neurobehavioral disturbances resulting from HIV-mediated neural damage include emotional and other behavioural disturbances (e.g. depression, anxiety, sleep disorders, mania and psychosis) [37].

### Diagnosis

Any individual with HIV infection could be at risk of developing HAND [38]. Therefore, the need for a systematic study of cognitive HIV infected individuals seems increasingly evident. Although many different studies assessing neurocognitive impairment in HIV infection have been published, a standard approach to optimally assessing neuropsychological deficits does not currently exist. Multiple clinical tests are available for use in order to evaluate neurocognitive function although there is currently no single and universal neuropsychological battery to assess specifically people with HIV. Evaluation of the different cognitive functions requires a concerted effort (2–4 hours) with the need for expert staff specialized in clinical neuropsychology who has to evaluate at least five of the following cognitive abilities: verbal/language, attention/working memory, abstraction/executive functioning, learning/recall, speed of informational processing, and sensory-perceptual and motor skills. This has led to the need for screening instruments that, although maybe useful in the initial screening, are insufficient to make a good neuropsychological diagnosis within clinics. The classic tests-mini-mental state examination (MMSE) or Montreal cognitive assessment (MoCA) are not sufficiently sensitive to screen for HAND unless the patients are symptomatic [39].

**Figure 1:**
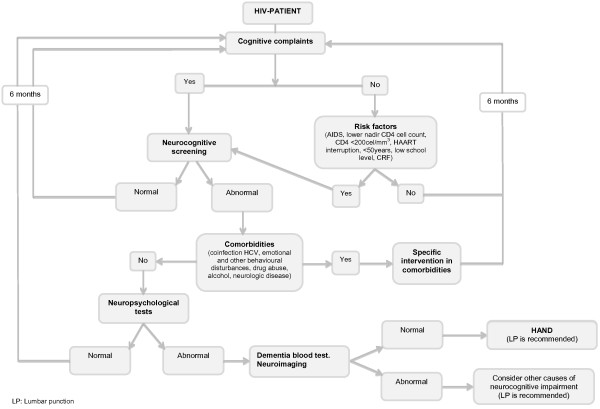
**Algorithm for the detection and evaluation of HAND.**

There are specific screening instruments for assessing the HIV Dementia Scale HAND (HDS) [40], *The International HIV Dementia Scale* (IHDS) [41] or the Montreal cognitive assessment (MoCA) [42]. All these tests can be performed in less than ten minutes. Other tests often used in conjunction with these include the *Brief Neurocognitive Screen* (BNC) [43], screening battery of HNRC [44], computed COGSTATE [45] or Neu Screening [46]. The diagnosis of HAND is confirmed by the clinical detection of cognitive impairment through neuropsychological testing and the evidence of neurocognitive impairment. Cognitive impairment must be attributable to HIV after excluding other causes that can justify the disorder (Figure 1).

### Differential diagnosis

The differential diagnosis of neurocognitive disorders associated with HIV infections presents with two difficulties: 1) to discard all the pathologies that can lead to neurocognitive disorders in the general population, associated or not with HIV; 2) to determine the degree of contribution of certain comorbidities associated with HIV infection, before attributing neurological damage to HIV itself. This group may act as true comorbidity confounders while attempting to diagnose a neurocognitive disorder in patients associated with HIV infection [47]. When the presence of neurocognitive impairment is suspected, an exhaustive study has to be done in order to detect other potential causes of neurocognitive impairment, including laboratory testing of vitamin B12, folate level, TSH, syphilis, hepatitis C, glucose and vitamin B1 as well as screening for dyslipidaemia. Sometimes, it is necessary to examine CFS especially when there are other signs of CNS infection, fever, CD4 cell counts below 200 cells/mm^3^ or positive serology for syphilis.

Other testing should include screening for major depression and anxiety, prescription of psycho-pharmacologic treatments and identification of self-abuse of other substances. Frequently, patients present other comorbidities such as psychiatric illness, vascular cerebral disease, opportunistic infections, alcohol and drugs abuse. All these factors can cause neurocognitive disorders or contribute to HAND progression [2]. It is a requirement to know the real impact of comorbidities in order to find new prevention and treatment strategies.

There is a growing interest in finding biomarkers that are sensitive and specific for HAND. One of the most reliable biomarker currently used for monitoring disease progression and HAART effectiveness is CD4 cell count. Counts above 200 cells/mL are rarely associated with dementia [48, 49]. Detection of HIV RNA concentration in the CSF can also be determined. The presence of HIV RNA in CSF, even at high concentrations, is not diagnostic for HAND and cognitively asymptomatic subjects may have high Viral Load (VL). In the CHARTER study, patients with CSF VL < 2.5 copies/mL had a higher degree of neurocognitive impairment compared to the ones with a VL of 2.5-40 copies/mL [50]. Some studies have shown slight hyper-proteinorraquia and pleocitosis with increased mononuclear counts [51]. This increased cellularity has been positively correlated with HIV viral load in CSF and can be reversed with proper antiretroviral treatment. However, these changes can also be found in subjects without cognitive impairment, so are not specific for diagnosis of HAND. In HIV infected patients, different biomarkers have been tested for their effectiveness, including amyloid peptide B, Tau and phospho-Tau [52]. HIV population studies show conflicting results. Some authors confirm that CSF abnormalities in HIV patients with HAD are similar to those from Alzheimer’s disease patients (increased tau and phospho-tau and decreased beta-amyloid peptide) [53] but still others do not support the idea [54]. A recent study, however, by Steinbrink et al., found a positive correlation between tau levels and the degree of HAND [55].

The existence of reliable biomarkers would help to diagnose patients and would allow better understanding of the pathophysiology of cognitive impairment in HIV patients, as well as designing therapeutic strategies aimed at treating complications. Although there are no pathognomonic neuroimaging alterations in HAND, it is pertinent to recommend neuroimaging for investigating other neurological disorders (opportunistic infections, tumours and other structural lesions or ischemia/infarction). RM is recommended and it is preferred to TAC because of its sensitivity [47]. Other techniques are being investigated to improve the diagnosis of HAND such as cerebral volumetry, PET, MRI and spectroscopy. They all are able to identify structural, metabolic and functional disorders which are related to the presence of cognitive disorder.

### Treatment

Since the main cause of cognitive impairment in HIV-infected patients is the virus infection itself, the treatment of choice (accepted treatment strategy) remains the use of HAART defined as a combination of three or more drugs. Clear improvement in neurological outcomes in the era of HAART has also been achieved resulting in a notable reduction of HAD. However, HAART benefits for HIV cognitive disorders differ substantially between individuals [56] and several studies have shown in recent years the persistence of asymptomatic or mild/moderate forms of HAND [57] leading to speculations that systemically effective HAART might fail to control CNS-HIV infection, and also that the type of antiretroviral drug and its penetration may have a role in the development of HAND. CSF HIV RNA is generally lower than in plasma and normally decays in parallel to plasma viral load when HAART is initiated. Therefore, the majority of patients with undetectable plasma VL have also undetectable CSF VL. However, in some cases CSF decay is delayed in response to HAART and approximately 10% of patients treated with HAART (for at least 6 months) have VL detectable in LCR despite suppression of plasma human HIV RNA [58]. It has been demonstrated that a greater viral suppression within the CNS is associated with a better neurocognitive function [59] and may influence survival [60]. Eden et al., found that patients with neurological disease had a significantly higher CSF viral load both at baseline and at follow-up compared with neurologically asymptomatic patients [61].

There is a considerable heterogeneity among different ARV agents with respect to their CNS penetration. A number of drug characteristics influence penetration across the BBB. The latter is composed of a complex of endothelial cells, transport proteins and basement membrane with fenestrated capillaries that together prevent passage of drugs and other substances into the brain and have the capacity to express molecular drug pumps or transports such as P-glycoprotein that can limit the amount of drug that passes into the brain. Letendre et al., created a CNS penetration effectiveness ranking system of different ARV agents based on chemical properties of the drugs (molecular weight, liposolubility, ion charge and its union with plasma proteins), data on CSF concentrations, and HAART effectiveness [62]. It was demonstrated that combination antiretroviral treatments with higher CNS penetration-effectiveness (CPE) achieved better CSF viral suppression [63] and could be associated with better cognition. There are no conclusive studies on the efficacy of HAART regimens regarding improvement of cognitive performance or which is the best combination to prevent or reduce cognitive impairment. In general, antiretroviral drugs have low molecular weight that favors crossing the BBB. The nucleoside analogue reverse transcriptase inhibitors (nRTIs) have a low molecular weight, followed by non nucleoside reverse transcriptase inhibitors (NNRTIs). The protease inhibitors (IPs) have clearly higher molecular weights and therefore show a low BBB penetration. In addition, their high affinity for P-glycoprotein makes it difficult to achieve high concentrations in the CNS. Ritonavir, a potent inhibitor of P-gp, improves CNS levels of other PIs when used as a booster [64]. Drugs with less affinity for P-gp penetrate the CNS more easily. PIs and NNRTIs exhibit greater fat solubility than nRTIs. Recently, new ARVs drugs have been approved and are also used in combination with other antiretroviral agents for treating HIV adult patients: Raltegravir, an integrase inhibitor which prevents viral DNA insertion into the human cell genome and Maraviroc, an antagonist of the CCR5 chemokine receptor, which also serves as an HIV-1 co-receptor. They have a lower plasma protein binding level than other ARVs and seem to be a substrate of P-gp transporter. Enfuvirtide is a fusion inhibitor which is indicated for HIV patients who have failed prior ART regimens. There are limited data on the CNS pharmacokinetics of this inhibitor. Finally, treatment regimens should be designed to optimize CNS penetration without sacrificing viral suppression or immunological improvement. Therefore a treatment regimen with good CNS penetration could be critical in the case of patients with disorders associated with neurocognitive function [65].

#### Other pharmacological treatments

Despite the introduction of HAART, the persistence of HAND has led to development of new strategy adjuvants to HAART. Multiple drugs with neuroprotective properties have been evaluated. Minocycline, a tetracyclin with neuroprotective properties and good penetration through the BBB is being evaluated as a possible adjunct to HAART. However, clinical trials performed on a cohort of 107 individuals have not demonstrated improvement in cognitive function [66]. Memantine, a non-competitive antagonist of N-methyl-D-aspartate, approved for treatment of Alzheimer’s disease has also been tested in a randomized double-blind, placebo-controlled, multicenter trial within the Adult AIDS Clinical Trials Group. No significant improvement in patients with mild to severe cognitive impairment after 16 weeks of treatment was noted. However, the magnetic resonance spectroscopy data suggested that memantine may ameliorate neuronal metabolism, an important step to stabilizing or preventing neuronal injury [67]. Other drugs have been investigated, including lithium, selegiline and valproate. At the present time, there is no data supporting the use of any adjuvant therapy to HAART for the treatment of neurocognitive disorders associated with HIV infection although some drugs such as memantine have been shown to improve markers of neuronal damage and inflammation within the CNS.

#### Non pharmacologic interventions

There is a little information regarding pharmacological interventions in HIV populations which can be extrapolated to the general population. It is essential to modify lifestyle (diet, physical activity, stress) and to give up tobacco and alcohol. Currently, we can prevent cardiovascular risk factors (e.g. HTA, DM, DLP). Adherence to treatment remains the central issue in HIV patients in order to keep control of VL. Neurocognitive impairment is strongly related to poor adherence [68]. The later can lead to drug resistance, increase morbidity/mortality and development of cognitive impairment, especially in the elderly [69]. Neuropyschological intervention programs proved to be useful in multiple pathologies like schizophrenia, acquired brain injury or Alzheimer’s disease. However, few studies had been performed in the HIV field. Some of the programs showed some positive effects [70] although proper studies with new strategies presently do not exist.

### Prognosis

Before the introduction of HAART, HIV associated neurocognitive impairment was considered a significant independent risk factor for AIDS related death. Although the median survival of patients with HAD has decreased consierably [71] it remains a negative prognostic factor associated with mortality [25, 72]. Other studies have shown that mild cognitive impairment is also associated with poorer adherence to treatment [73], higher rates of virological failure [74], difficulties in the development of everyday function [75], los of employment [76], impairments in the quality of life [77] and progression to dementia [78]. In the post-HAART era, the course of dementia progression has become more heterogeneous and appears to be related to factors such as antiretroviral therapy adherence, aging, substance abuse and psychosocial factors. Evolution is unknown in patients who have cognitive impairment. Few studies have been conducted to assess the incidence and persistence of HAND despite HAART. A study of cohorts which evaluated a group of 94 patients with HIV-related neurocognitive impairment, found that to 63% of them, had persistent neurocognitive deficits within 63 months of successful treatment. Also, 37% had permanent deficits without alterations in subsequent visits. They concluded that the severity of neurocognitive impairment at HAART initiation seems to be the strongest predictor of persistent neuropsychological deficits [26]. In another cohort of 1561 HIV+ patients the presence of HAND was associated with a 3-fold increase in mortality [79].

In recent years the concept of “brain reserve” (CR) has emerged to describe some individuals having an increased “baseline adaptive neuroplasticity”, providing greater dynamic capacity for adjusting and remodeling cortical circuits to various stressors [80]. Another neuropsychiatric symptom that results from CNS viral damage is apathy is significantly associated with cognitive impairment in individuals with HIV; Previous literature has demonstrated CR’s capacity to protect against cognitive decline and apathy in individuals in HIV-infected individuals across the age span, particularly after a certain threshold of disease severity [81].

## Conclusions

Currently, neuropsychological impairment among HIV+ patients on ARV therapy leads to a reduction in the quality of life and it is an important challenge due to the high prevalence of HAND and its concomitant consequences in relation to morbidity and mortality- including those HIV+ patients with adequate immunological and virological status. The fact that the virus is established in CNS in the early stages and its persistence within the CNS can help us to understand HIV-related brain injury even when HAART is effective. Longitudinal studies focussing on neuropsychological function are needed, as there is a few data on progression and clinical significance of ANI in these patients. In addition, we have little information about risk factors or markers of progression of HAND. The most important risk factors associated with neurocognitive impairment have been described in cross-sectional studies or non-representative sample, and very often with contradictory results. It is essential to address comorbidities in patients with cardiovascular risk factors or depression. The rising interest in HIV associated neurocognitive disorders has led to development new diagnostic tools, improvement of the neuropsychological tests, and the use of new biomarkers and new neuroimaging techniques that can help the diagnosis. Standardization and homogenization of neurocognitive tests as well as normalizing and simplification of easily accessible tools to screen for patients with increased risk of cognitive impairment and prioritize full neurocognitive study is an urgent requirement. The short screening instruments would be more important in places where it is not possible to complete full study. For the moment, there is not a universal recommendation how to proceed in clinical practise. It is strongly recommended to ask patients routinely for cognitive symptoms as detection of neurocognitive impairment can justify starting ARV therapy. It is also important to make a correct monitoring of cognitive complaints on every visit.

Finally, standard treatment for HIV-associated neurocognitive disorders is an optimal antiretroviral treatment regimen, along with control and treatment of other comorbidities or psychiatric problems. For naïve HIV+ patients with evidence of cognitive deficits, it is recommended to start ART with an optimized central nervous system penetration effectiveness rank. However, pharmacodynamic studies of HAART are needed in order to determine which drugs can be more effective in preserving cognitive functioning and when it is necessary to change the treatment if cognitive impairment appears, even when virological control has been achieved. Future efforts should also focus on diagnostic keys and searching for useful strategies in order to decrease HIV neurotoxicity within the CNS.
